# The correlations on psychopathology in children self-rating, psychopathology in children as related by their parents and psychopathology in parents self-rating in a Kenyan school setting: towards an inclusive family-centered approach

**DOI:** 10.1186/s12888-024-05971-1

**Published:** 2024-07-25

**Authors:** David M. Ndetei, Victoria Mutiso, Pascalyne Nyamai, Christine Musyimi

**Affiliations:** 1 Africa Institute of Mental and Brain Health (Formerly Africa Mental Health Research and Training Foundation), Nairobi, Kenya; 2 Department of Psychiatry, Kenya and Founding Director of Africa Institute of Mental and Brain Health (Formerly Africa Mental Health Research and Training Foundation (AMHRTF)), University of Nairobi, Mawensi Road, Off Elgon Road, Mawensi Garden, P.O. Box 48423-00100, Nairobi, Kenya; 3 World Psychiatric Association Collaborating Centre for Research and Training, Nairobi, Kenya

**Keywords:** Psychopathologies, Children, Parents, Kenya, ASR, YSR, CBCL

## Abstract

Several studies have reported on the association between parental and childhood psychopathologies. Despite this, little is known about the psychopathologies between parents and children in a non-clinical population. We present such a study, the first in a Kenyan setting in an attempt to fill this gap. The objective of this study was to determine the association between self-rating psychopathology in children, parent-rating psychopathology in their children and self-rating psychopathology in parents in a non-clinical population of children attending schools in Kenya. We identified 113 participants, comprising children and their parents in 10 randomly sampled primary schools in South East Kenya. The children completed the Youth Self-Report (YSR) scale and parents completed the Child Behavior Check List (CBCL) on their children and the Adult Self-Reports (ASR) on themselves. These instruments are part of the Achenbach System of Empirically Based Assessment (ASEBA), developed in the USA for a comprehensive approach to assessing adaptation and maladaptive behavior in children and adolescents. There was back and forth translation of the instruments from English to Swahili and the local dialect, Kamba. Every revision of the English translation was sent to the instrument author who sent back comments until the revised version was in sync with the version developed by the author. We used the ASEBA in-built algorithm for scoring to determine cut-off points for problematic and non-problematic behavior. Correlations, linear regression and independent sample t-test were used to explore these associations. The mean age of the children was 12.7. While there was no significant association between child problems as measured by YSR (self-reported) and parent problems as measured by ASR and CBCL in the overall correlations, there was a significant association when examining specific groups (clinical range vs. non-clinical). Moreover, significant association existed between total problems on YSR and ASR internalizing problems (*t=-2.3*,*p* = 0.023), with clinical range having a higher mean than the normal range. In addition, a significant relationship (*p* < 0.05) was found between psychopathology in children as reported by both parents (CBCL) and psychopathology in parents as self-reported (ASR).

Mothers were more likely to report lower syndrome scores of their children as compared to fathers. Our findings indicate discrepancies between children self-rating and parent ratings, suggesting that one cannot manage psychopathology in children without reference to psychopathology in their parents. We suggest broad-based psycho-education to include children and parents to enhance shared awareness of psychopathology and uptake of treatment.

## Introduction

A multi-country study (which did not include Africa) involving 61,703 parents ratings and 29,486 youth self-ratings, concluded that Child Behavior Check List (CBCL) 6–18 and Youth Self-Report (YSR) syndromes offer clinicians psychometric robust ways to conceptualize problems reported for children in diverse backgrounds [1]. Better parent-adolescent agreement and more consistency in agreement across diverse societies are found in the clinical samples than in population samples [2]. While there are many cross-information consistencies on CBCL and YSR across countries there are also some differences in parent-adolescent cross-information agreement [3, 4].

The differences in parent-adolescent cross-information agreement across countries could be explained by contextual factors either within the parent or within the child, for example, substance use, depression and low school motivation in the children or parental depression, stress, low family income and family dysfunction [5–8]. For instance, a Greek study found that parents with higher levels of psychopathology reported higher levels of psychopathology in their children compared to the children’s self-reports. Additionally, lower family income and lower parental education levels were associated with greater discrepancies [6]. Maternal levels of psychopathology predict adolescents’ externalizing problems [9–13], and fathers’ depression, as evidenced by previous research, is associated not only with children’s externalizing problems but also with their internalizing problems (emotional problems) [14]. Additionally, stigma surrounding mental health and differences in access to healthcare can impact informant reports in diverse cultural settings. In Western countries, where people are more outspoken about mental health and there is less stigma, informant reports may differ from those in African countries, where the stigma surrounding mental health issues remains prevalent [15–17].

Most studies have dealt with mothers and children psychopathology while a few considered fathers and children psychopathology [18]. It is important to look at child psychopathology using different informants, such as self-reports from children (YSR) and reports from parents (CBCL), because this will give a comprehensive assessment of child mental health. Parents and children may perceive the child’s behaviors and symptoms differently. This is especially important in the Kenyan context, where such studies are lacking thus helping in informing more accurate diagnoses and interventions.

In a situation where the overwhelming majority of youth do not access clinical services either because they are not available or the parents do not recognize the symptoms at all or may not even think they represent a mental disorder, the majority of such youth do not get appropriate attention. There is therefore the need to strategize on an approach that identifies them at the earliest possible time while they are still in the communities or schools. This will facilitate a critical reach and therefore a necessary intervention. This study hopes to demonstrate this by studying children not yet attending clinical services. Equally and critically important, until you bring the children, parents, and teachers to a common understanding of psychopathology through dialogue, the old practice will continue, the biggest casualty being the child who feels misunderstood. This approach is an important move towards effective dialogue, bringing in all the key players.

One of the most widely used instruments for documenting psychopathology in children as perceived by the children themselves and as perceived by their parents, and psychopathology in parents as perceived by themselves is the *ASEBA* [19]. According to this instrument, internalizing problems are viewed as behaviors and emotions directed inwards which include; anxiety, somatic and depressive symptoms, while externalizing problems are conceptualized as behaviors and emotions directed outwards which include: aggressive, oppositional and rule-breaking behaviors. Internalizing problems in both mothers and fathers are associated with internalizing problems in their children and externalizing problems in both mothers and fathers are associated with externalizing problems in their children [20, 21].

## The current study

While numerous studies in Western countries have explored child psychopathology using both clinical and community samples [22–26], there is paucity of such research in Kenya and other African contexts. In this study in a Kenyan social-cultural context we seek to answer the following research questions: [1] Is there a correlation between YSR by children and CBCL by parents on the children (YSR/CBCL scores versus ASR (Adult Self-Reports) on the adults) [2]? Do parents with psychopathology over-report or under-report psychopathology in their children? Answering these questions in primary school children is important – i.e. how YSR/CBCL scores compare with ASR. Focusing on upper primary school children (ages 11–14), who form 30.9% of the Kenyan youth [27] is essential as they are in the early stages of adolescence and beginning to develop self-identify. Understanding how YSR/CBCL scores compare with ASR scores in this significant age group will provide valuable insights into the correlation between child and parent reports and the potential impact of parental psychopathology on these reports. There is therefore the need to understand the dynamics of psychopathology in school going children and psychopathology in parents. This will inform person and family centered intervention.

Further to what is explained earlier on why non-clinical population, studying a non-clinical population is critical especially in Kenya where according to the Kenya Mental Health Policy 2015–2030, the government is aiming at ensuring that all persons have access to comprehensive, integrated, and high-quality mental health care that is promotive, preventive and curative [28]. This can be achieved by integrating mental health into primary healthcare. This study will also provide valuable data that can help practitioners better understand the mental health needs of Kenyan children thus supporting the practitioner in developing targeted interventions. The study will make contributions to the global literature and database as well as enhance the global evidence base, informing cross-cultural comparisons and contributing to the development of universally applicable mental health interventions. In summary: - [1] We will provide data that are based on non-clinical populations that are least reported in the literature; [2] We will provide vital context-appropriate information for Kenyan practitioners – whether in clinical services or in public health awareness campaigns on how to relate psychopathologies in parents and children. This information could find application in other similar social-cultural contexts outside Kenya; [3] The generated data will contribute to the global data on the same subject.

### The general objective

The general objective of the study is to determine the correlations on psychopathology in children self-rating, psychopathology in children as rated by their parents and psychopathology in parents self-rating in a Kenyan School Setting.

### The specific aims

The specific aims of the study are: [1] to determine the correlation between YSR by children and CBCL by parents on the children (YSR/CBCL scores versus ASR (Adult Self-Reports) on the adults); [2] to determine if parents with psychopathology over-report or under-report psychopathology in their children.

## Methods

### Participants and study site

This study was part of a bigger study titled “The Kenya Integrated Intervention Model for Dialogue and Screening to Promote Children’s Mental Wellbeing (KIDS)” [29]. The participants (non-clinical) were drawn from ten schools in Machakos Sub-Country in South East Kenya. In order to facilitate effective administrative supervision of schools by the school supervisors, the schools in Machakos sub-county are divided into administrative groups; each group is referred to by the Ministry of Education (MoE) as a Cluster. To meet our predetermined sample, we randomly chose ten clusters and then randomly selected one school per cluster. This sampling procedure had been used successfully in another study but excluded the ten schools used in this study [30]. We approached the ten primary schools and explained the nature of the study to the head teachers for their permission to undertake the study in the respective schools, followed by the school boards and the Parents Teachers Association (PTA).

Respondents were recruited from their respective classes (Class 5–8; aged 11–14). For each class, in the ten schools, potential participants were randomly selected. Based on the average number of students per class, a sampling interval was calculated using this formula: $\:\varvec{k}=\frac{\varvec{N}}{\varvec{n}}$ where; k = sampling interval, N = population of children in a class, and n = number of children to be sampled. Our sample size per class was informed by the fact that this was part of a bigger study in which we also included teachers’ perceptions of psychopathology in their students. However, the focus of this study was psychopathology in the children and psychopathology in both male and female parents and their condition.

Every k^th^ individual who was sampled was recruited into the study until the minimum sample was met. Whenever a child was selected for the study and the parent was not available/ to give consent, the next child whose parent was available and consented to be part of the study was selected. The class teacher could rate up to four of their students without fatigue on the day the parents were available. This translated to about 11 students per school and therefore each class about three students.

We then asked for the parent’s consent for their children to participate in the study. The children were informed about the nature of the study and asked for their assent to participate in the study. Only parents and children from whom we had consented and assented were included in the study.

### Ethics consideration

#### Ethics approval

was obtained from the Kenya Medical Research Institute (KEMRI) SERU (Non-SSC Protocol No. 383) and the Centre for Addiction and Mental Health Research Ethics Board, Canada, protocol reference number is #194/2013. All methods were performed in accordance with the relevant guidelines and regulations. Informed consent/assent was obtained from all subjects and/or their parents/legal guardian(s).

### Procedure

We undertook 2-day training for 20 research assistants (RAs), 2 per school, on how to administer our research instruments i.e. YSR, CBCL and ASR. The training included reading through all the questions in a group and administering the questions to one another until there was uniformity in how the RAs read each of the questions. They were trained to read the questions on ASR and CBCL to a parent up to 3 times without any elaboration and then record the answer. If the parent still did not understand by the third time, the RAs were to skip that question. The RAs administered the instruments after checking the validity of all the consents and assents. Each child completed YSR using the adapted English version since English is their language of instruction. Only children who completed YSR and their parents who completed CBCL and ASR were included in the study. All the parents opted to complete CBCL and ASR in the local dialect (Kamba) as was read to them by RA as detailed above.

### Measures

There were two sets of instruments: [1] a demographic questionnaire in which the RA noted the gender of the child, asked for the class of the child and the RA also noted the gender of the parent and asked for their age. [2] Psychometric instruments – CBCL, YSR and ASR. The process of adaptation of the instruments (to ensure original meaning was retained; back and forth translation from English to Swahili and the local dialect, Kamba, piloting and adoption) has already been reported for CBCL and YSR [30, 31]. In summary, this translation was done in consultation with the authors of ASEBA to satisfy them that the back-translated versions reflected the original meaning and concepts behind the original questionnaires developed by them. We, therefore, used back translations that were approved by the authors of ASEBA to ensure that they were at par with all other translations from various languages and cultural backgrounds. All scoring was done by a standardized algorithm software used in all studies using these tools.

The specific measurement tools used in this study (CBCL, YSR and ASR) have not been formally validated in Kenya. However, the use and reported internal consistency of these tools in other sub-Saharan African countries provide a reasonable basis for their application in this study [32]. Additionally, studies in diverse populations have shown these measures to be reliable and valid for assessing child psychopathology [33].

The CBCL measures internalizing and externalizing behavior problems in children as perceived by the parents [19]. The questionnaire contains 113 items which are rated on a 3-point Likert scale (0 = ‘not true’, 1 = ‘sometimes true’ or ‘somewhat true’, 2 = ‘often true’ or ‘very true’). CBCL also measures attention problems, an important aspect of child behavior. However, this study focused on specific domains of psychopathology that were deemed most relevant to our research aims. We thus prioritized internalizing and externalizing problems as our main areas of investigation and recommend that attention problems can be a subject for another study. The YSR is a self-report measure completed by children aged 11–18 to assess emotional and behavioral problems. The CBCL and YSR subscales are relatively comparable in the domains they measure. Both instruments are designed to assess similar aspects of child behavior and emotional problems, including internalizing and externalizing symptoms.

Both the CBCL and YSR have demonstrated good to excellent consistency ranging from 0.50 to 0.82 [34], and have good psychometric properties with high levels of internal consistency (Cronbach’s alpha = 0.82 for DSM-IV oriented scales), as well as high test-retest reliability (*r* = 0.88) [35], *r* = 0.97 test-retest reliability for CBCL [36].

The ASR is a 126-item parent self-rating tool that has good internal consistency for most scales, with mean alpha coefficients on the ASR and ABCL of 0.83 and 0.85 for the empirically based problem scales and 0.78 and 0.79 for the DSM-oriented scales, and test-retest reliability of between 0.80 and 0.90 [19, 37]. It is completed by either the mother or father so as to determine their internalizing and externalizing problems.

### Data management

The data from the YSR, CBCL and ASR were double entered and scored by the Assessment Data Manager (ADM) software version 9.1, a tool developed by the Achenbach System of Empirically Based Assessment (ASEBA) team [33]. The ADM scores each assessment and produces a summation of all problem items and ratings for the DSM-IV Oriented Scale separately for boys and girls and separately with raw, standardized and percentile scores. Raw scores, as well as the demographic data, were converted into SPSS through A2S (a one-way utility designed to process data from Assessment Data Manager (ADM) or Ratings-to-Scores (RTS) into SPSS already scored).

### Data analysis

Statistical analysis was done with the view to achieving our aims. To achieve the first aim, we used continuous YSR, CBCL and ASR scores in our main analyses, given the evidence indicating that the intensity of psychopathology (internalizing, externalizing and the total problem of the parent rather than meeting categorical criteria for clinical diagnosis) is a sensitive predictor of child outcomes [38]. First, bivariate correlations were used to examine associations between child psychopathology and parent psychopathology, followed by linear regressions to test the unique relationship between parent psychopathology (predictors) and child psychopathology (outcomes). Covariates, including the age of the child and the parent, as well as the gender of the child and the parent, were included. In the first step, parents’ internalizing, externalizing, and total problems were entered simultaneously into the model, while in the second step, the age and gender of both the child and the parent were included in the model. For the second aim, we used a t-test to test whether parents of children classified in the clinical range had higher psychopathology than those with children in the normal range. To address multicollinearity, we examined Variance Inflation Factor (VIF) values. While most predictors had VIF coefficients below 10, only internalizing and total problems of parents exceeded this threshold. We addressed this by analyzing them separately in regression models. All the analyses were conducted by SPSS version 23, and the level of significance was set at alpha < 0.05. All tests were two-sided. To determine the cut-off points for clinical and non-clinical level scores we used the same cut-off points used by ASEBA and which have been validated in several cross-cultural studies [39]. The use of ASEBA cut-off points in studies that involve cultural contexts similar to Kenya provides preliminary evidence supporting the use of these cut-off points in our study.

## Results

### Response rate

Out of all the potential participants approached, only one parent did not respond.

All the 113 children identified and approached and their parents participated and completed the study with only one parent not completing the study, giving a response rate of 99.1% (Table 1). These results are divided into 3 categories: [1] The demographical variables (Table 1); [2] Results that achieved Aim 1 (Tables 2, 3, 4 and 5); [3] Results that achieved Aim 2 (Table 6).

**Table 1:** Demographic characteristics of the respondents

		*N* = 113	
Variable	Category	Frequency	Percentage (%)
Gender of the Child	Female	57	50.4
	Male	56	49.6
Class of the Child	Class 5	22	19.5
	Class 6	25	22.1
	Class 7	35	31.0
	Class 8	31	27.4
Gender of the Parent	Female	60	53.6
	Male	52	46.4
	*Missing*	1	
**Variable**	**Mean**	**SD**	**Range**
Age of the Child	12.7	1.1	11–14
Age of the Parent	39.0	6.2	29–61

The table displays the frequency and percentage distribution of children’s gender, class, and parents’ gender, as well as the mean, SD: Standard deviation, and range of ages

**Table 2:** Correlations between children’s problem scores (self-reported YSR) and parent’s problem scores on themselves (ASR)

Part-A (ASR vs. YSR)		*N* = 113		
ASR		Internalizing problems Child (YSR)	Externalizing Problems Child (YSR)	Total Problems Child (YSR)
Internalizing problems (ASR)	Correlation	**-0.034**	**0.008**	**0.003**
	Sig. (2-tailed)	0.722	0.932	0.976
Externalizing Problems (ASR)	Correlation	**0.008**	**0.129**	**0.074**
	Sig. (2-tailed)	0.937	0.175	0.435
Total problems (ASR)	Correlation	**-0.007**	**0.057**	**0.036**
	Sig. (2-tailed)	0.943	0.551	0.707

Pearson correlations between YSR and ASR across internalizing, externalizing, and total problems. Bold Correlation values

**Table 3:** Correlations between parents reported problems on children (CBCL) and parent’s problem scores on themselves (ASR)

		*N* = 113		
		Internalizing problems Child (CBCL)	Externalizing Problems Child (CBCL)	Total Problems Child (CBCL)
Internalizing problems (ASR)	Correlation	**-0.052**	**-0.065**	**-0.042**
	Sig. (2-tailed)	0.587	0.496	0.656
Externalizing Problems (ASR)	Correlation	**-0.096**	**-0.101**	**-0.097**
	Sig. (2-tailed)	0.313	0.287	0.304
Total problems (ASR)	Correlation	**-0.057**	**-0.061**	**-0.048**
	Sig. (2-tailed)	0.548	0.523	0.616

Pearson correlations between CBCL and ASR across internalizing, externalizing, and total problems. Bold Correlation values

**Table 4:** Comparison of clinical range problems for the children (YSR and CBCL) and parent syndrome scores (ASR) (^‡^See footnote)

Problem	Diagnosis	*N* = 113 (100%)	Internalizing Problems (ASR)	t-test on group differences	Problem	Diagnosis		*N* = 113	Internalizing Problems (ASR)	t-test on group differences
			Mean ± S.D						Mean ± S.D	
YSR					CBCL					
Internalizing Problems (YSR)	Normal	75 (66.4)	19.1 ± 12.2	t_(111)_=-0.09, *P* = 0.931	Internalizing (CBCL)	Normal	61(54.0)		14.6 ± 11.8	t_(111)_=-4.82, **P<0.001**
	Clinical	38(33.6)	19.3 ± 11.9			Clinical	52(46.0)		24.6 ± 10.0	
Externalizing Problems(YSR)	Normal	104(92.0)	19.1 ± 11.9	t_(111)_=-0.21, *P* = 0.837	Externalizing (CBCL)	Normal	96(85.0)		17.1 ± 10.7	t_(111)_=-4.74, **P<0.001**
	Clinical	9(8.0)	20.0 ± 13.6			Clinical	17(15.0)		30.9 ± 12.5	
Total Problems(YSR)	Normal	99(87.6)	18.2 ± 11.8	t_(111)_=-2.3, **P<0.023**	Total Problems CBCL	Normal	82(72.6)		16.3 ± 11.7	t_(111)_=-4.54, **P<0.001**
	Clinical	14(12.4)	26.0 ± 12.0			Clinical	31(27.4)		26.9 ± 9.4	
**Problem**	**Diagnosis**	**N**	**Externalizing Problems** **(ASR)**						**Externalizing Problems** **(ASR)**	
Internalizing Problems (YSR)	Normal	75 (66.4)	11.3 ± 8.6	t_(111)_ = 0.31, *P* = 0.754	Internalizing (CBCL)	Normal	61(54.0)		9.4 ± 8.3	t_(111)_=-2.65, **P = 0.009**
	Clinical	38(33.6)	10.8 ± 6.8			Clinical	52(46.0)		13.3 ± 7.2	
Externalizing Problems (YSR)	Normal	104(92.0)	11.2 ± 8.3	t_(111)_ = 0.15, *P* = 0.877	Externalizing (CBCL)	Normal	96(85.0)		9.7 ± 7.0	t_(111)_=-5.15, **P<0.001**
	Clinical	9(8.0)	10.8 ± 4.4			Clinical	17(15.0)		19.5 ± 8.8	
Total Problems(YSR)	Normal	99(87.6)	11.0 ± 8.3	t_(111)_=-0.59, *P* = 0.560	Total Problems CBCL	Normal	82(72.6)		9.6 ± 7.7	t_(111)_=-3.63, **P = < 0.001**
	Clinical	14(12.4)	12.4 ± 5.5			Clinical	31(27.4)		15.4 ± 7.4	
**Problem**	**Diagnosis**	**N**	**Total Problems** **(ASR)**						**Total Problems** **(ASR)**	
Internalizing Problems(YSR)	Normal	75 (66.4)	48.7 ± 30.7	t_(111)_ = 0.31, *P* = 0.758	Internalizing (CBCL)	Normal	61(54.0)		38.6 ± 29.5	t_(111)_=-4.04, **P<0.001**
	Clinical	38(33.6)	46.9 ± 25.4			Clinical	52(46.0)		59.3 ± 24.0	
Externalizing Problems(YSR)	Normal	104(92.0)	48.2 ± 29.1	t_(111)_ = 0.12, *P* = 0.908	Externalizing (CBCL)	Normal	96(85.0)		42.5 ± 24.6	t_(111)_=-5.50, **P<0.001**
	Clinical	9(8.0)	47.0 ± 28.0			Clinical	17(15.0)		79.8 ± 32.0	
Total Problems(YSR)	Normal	99(87.6)	46.6 ± 29.2	t_(111)_=-1.43, *P* = 0.157	Total Problems CBCL	Normal	82(72.6)		41.0 ± 27.7	t_(111)_=-4.57, **P<0.001**
	Clinical	14(12.4)	58.4 ± 25.7			Clinical	31(27.4)		66.7 ± 23.7	

Table presents problem diagnosis frequencies and corresponding mean scores with standard deviations, along with t-test results for group differences ^‡^ASR does not provide for classification into normal and clinical ranges

**Table 5:** Multivariate analysis on Association between Childhood psychopathology (self-reported -YSR) and parental psychopathology (ASR)

	Internalizing Problems-YSR				Externalizing Problems-YSR				Total Problems-YSR			
	Crude		Adjusted		Crude		Adjusted		Crude		Adjusted	
**A**	β (s.e)	p-value	β (s.e)	p-value	β (s.e)	p-value	β (s.e)	p-value	β (s.e)	p-value	β (s.e)	p-value
Internalizing Problems-Parent	-0.028 (0.08)	0.719	-0.085(0.11)	0.425	0.006 (0.07)	0.932	-0.140(0.10)	0.172	0.007 (0.24)	0.976	-0.337(0.34)	0.317
Externalizing Problem-Parent	0.009 (0.12)	0.936	0.059(0.15)	0.700	0.152 (0.11)	0.168	0.259(0.15)	0.080	0.289 (0.37)	0.430	0.501(0.48)	0.303
Girl			1.864(1.89)	0.327			0.909(1.82)	0.619			2.484(6.01)	0.680
Mother			4.304(2.91)	0.142			4.519(2.80)	0.110			15.843(9.24)	0.089
Age parent			-0.005(0.16)	0.973			0.016(0.16)	0.916			0.222(0.51)	0.666
Age Child			-1.444(0.87)	0.098			-0.949(0.83)	0.258			-5.080(2.75)	0.067
**B**												
Total problems-parent	-0.002 (0.03)	0.942	-0.012(0.03)	0.710	0.019 (0.03)	0.547	0.010(0.03)	0.763	0.039 (0.10)	0.704	0.000(0.10)	1.000
Girl			2.016(1.88)	0.285			1.259(1.83)	0.493			3.253(5.98)	0.588
Mother			3.808(2.83)	0.181			3.607(2.76)	0.194			13.723(9.02)	0.131
Age parent			-0.015(0.16)	0.923			-0.002(0.16)	0.990			0.179(0.51)	0.727
Age Child			-1.439(0.86)	0.098			-0.965(0.84)	0.254			-5.100(2.75)	0.067

The table presents results of multivariate analysis on the association YSR and ASR, including crude and adjusted β coefficients and p-values for various factors. β: Beta coefficients and standard errors (s.e). A- Predictors: Parent Internalizing and externalizing problem: Covariates: Child and Parents Gender and Age. B- Predictor: Parent Total problem: Covariates: Child and Parents Gender and Age *Note* We opted not to include class level as a covariate in our linear regression model because there is an extremely high correlation between class level and child’s age (*r = 1*). Including both variables would lead to multicollinearity issues, which could distort our model’s results

**Table 6:** Multivariate analysis on Association between Childhood psychopathology parent reported (CBCL) and parental psychopathology (ASR)

	Internalizing Problems-CBCL				Externalizing Problems-CBCL				Total Problems-CBCL			
	Crude		Adjusted		Crude		Adjusted		Crude		Adjusted	
**A**	β (s.e)	p-value	β (s.e)	p-value	β (s.e)	p-value	β (s.e)	p-value	β (s.e)	p-value	β (s.e)	p-value
Internalizing Problems-Parent	-0.039 (0.07)	0.583	0.053 (0.10)	0.597	-0.039 (0.06)	0.491	0.048 (0.08)	0.530	-0.088 (0.20)	0.652	0.240 (0.27)	0.372
Externalizing Problem-Parent	-0.110 (0.11)	0.306	-0.138 (0.14)	0.339	-0.090 (0.08)	0.281	-0.107 (0.11)	0.334	-0.303 (0.29)	0.298	-0.441 (0.39)	0.256
Girl			-1.271 (1.78)	0.477			-0.679 (1.37)	0.622			1.057 (4.80)	0.826
Age Child			-4.607 (2.74)	0.096			-0.444 (0.63)	0.48			1.492 (2.19)	0.498
Mother			-0.008 (0.15)	0.957			5.184 (2.11)	**0.015**			17.791 (7.38)	**0.018**
Age parent			-0.053 (0.82)	0.948			-0.076 (0.12)	0.516			-0.206 (0.41)	0.616
**B**												
Total problems-parent	-0.018 (0.03)	0.543	-0.009 (0.03)	0.776	-0.015 (0.02)	0.518	-0.003 (0.02)	0.911	-0.041 (0.08)	0.611	-0.001 (0.08)	0.987
Girl			1.426 (1.77)	0.422			0.808 (1.36)	0.554			1.647 (4.77)	0.731
Age Child			-0.038 (0.81)	0.963			0.459 (0.63)	0.466			1.542 (2.20)	0.484
Mother			4.279 (2.67)	0.112			4.931 (2.05)	**0.018**			16.489 (7.20)	**0.024**
Age parent			-0.003 (0.15)	0.987			-0.073 (0.12)	0.535			-0.185 (0.41)	0.651

The table presents results of multivariate analysis on the association YSR and ASR, including crude and adjusted β coefficients and p-values for various factors. β: Beta coefficients and standard errors (s.e). A- Predictors: Parent Internalizing and externalizing problem: Covariates: Child and Parents Gender and Age. B- Predictor: Parent Total problem: Covariates: Child and Parents Gender and Age *Note* We opted not to include class level as a covariate in our linear regression model because there is an extremely high correlation between class level and child’s age (*r = 1*). Including both variables would lead to multicollinearity issues, which could distort our model’s results

### Demographical variables

These are summarized in Table 1. The sample consisted of 113 children comprising female 57 (50.4%) and male 56 (49.6%). The child’s age averaged 12.7 years (SD = 1.1, range = 11–14), while the parent’s averaged 39 years (SD = 6.2, range = 29–61).

### Aim 1

Table 2 summarizes the correlations between YSR (Self-reported) Vs. ASR (Self-reported) mean scores. The correlation coefficient between child internalizing problems (YSR) and parents internalizing problems (ASR) (-0.034), and child internalizing problems (YSR) and parents total problems (ASR) (-0.007) were the only variables with negative correlations. Table 3 summarizes the correlations between CBCL means scores and CBCL (Parent-reported) Vs. ASR (adult self-reported). They all had a weak negative correlation. There was no significant association between the child (Self-reported-YSR and Parent reported-CBCL) problems and parent problems (ASR) in all the correlations.

Table 4 summarizes the results of the comparison between children’s problems in the clinical range (self-reported YSR and parent-reported CBCL) and parent’s syndrome scores. There was a significant relationship (*p* < 0.05) between psychopathology in the children as reported by the parents (CBCL) and psychopathology in the parents as self-reported by the parents (ASR), with higher mean scores in the clinical range than the normal range. However, such association did not exist between YSR, as reported by the children themselves, and ASR except for total problems with YSR and ASR internalizing problems, with the clinical range (*M = 26.0*) having a higher mean than the normal range (*M = 18.2*). This difference was statistically significant (*t=-2.3*,*p = 0.023*).

Table 5 summarizes the results of Multivariate Multiple Regression Analysis of the Association between Childhood Psychopathology (Self-reported -YSR) and Parental Psychopathology (ASR). After adjusting for all other variables, no significant association was found (*p* > 0.05) between child internalizing; externalizing; and total problems and parents problem scores.

### Aim 2

Table 6 summarizes the results of Multivariate Multiple Regression Analysis of the Association between clinical and sub-clinical psychopathology in children and parents. After adjusting for all other variables, no significant associations were found (*p* > 0.05) between child internalizing; externalizing; and total problems and parents problem scores. There was a significant association between parent-reported externalizing and total problems of the child and the gender of the parent, where mothers reported significantly lower levels as compared to the fathers (*p* < 0.05).

## Discussion

The almost 100% (99.1%) response rate is not surprising in Kenyan community-based studies where communities are keen to understand mental illness and secondly, because of our community engagement approach [40, 41]. It was pleasantly surprising that up to nearly 47% male parents participated in this study. In a way, our study could well be among the first reported studies where there was almost equity in parental gender, as most of the studies have reported mother participation almost to the exclusion of fathers [12]. We attribute this almost gender equity to our engagement with the parents through the parents teachers association and involvement of the parents at their homes through their children, in which we informed them that the exercise was in the best interest of not only their children but also both parents. A general finding was that age and gender of the children did not influence syndrome scores as demonstrated by the results from our multivariate analysis.

This study reports, for the first time in a Kenyan context, the association of current psychopathology in parents using ASR and current psychopathology in children as perceived by the children themselves using YSR and as perceived by their parents using CBCL. Even as we discuss these associations there is the caveat of the possibility that parents with psychopathology have reporting biases for their children’s psychopathology in which they may perceive psychopathology in their children from the perspective of their psychopathology. Our discussion is structured into two sections, aligned with the two research aims.

1. **YSR vs. CBCL correlation**: The lack of correlation between YSR and CBCL scores on internalizing, externalizing and total problems, with a *p*-value of > 0.05, confirms other studies to the effect that children tend to see themselves differently from how the parents see them and vice versa [42]. This implies that parents and children may perceive and report on behavior differently, even when assessing the same individual. Factors such as communication barriers, differing perspectives on behavior, and parental biases could contribute to discrepancies between parents reports and youth self-reports. The clinical implication is that there is a need in the Kenyan situation to take a family-orientated approach to the management of children with mental disorders. This approach may bridge the gap between the children understanding of their problems and that of the parents understanding of the problems in their children.
2. **YSR/CBCL vs. ASR**: The overall less agreement between ASR and YSR and between ASR and CBCL on the general range of symptom scores found in this study is at variance with most reported studies, most of which were on clinical population. This can be explained in that nearly all the studies referenced in the introduction were on clinical populations of both children and parents, whereas in this study, they were in a non-clinical community-based setting who were treatment-naive and presumably many did not have symptoms achieving clinical significance. However, our results concur with the findings of studies on clinical populations when we classify the children into non-clinical and clinical categories and then compare the syndrome scores with those of the parents themselves. We found out that parents of children within the clinical range have significantly higher syndrome scores of ASR as compared to parents with children in the normal range on CBCL clinical categories (*p* < 0.05). The only significant association between YSR and ASR was in the clinical total scores (*p* = 0.023), a likely carryover of externalizing problems and in particular ADHD which is characterized by highly visible disruptive behaviour and for which there is a tendency for children, parents and teachers to agree on. Overall, our findings concur with general trends that children rarely agree with parents on self-rated psychopathology.

A possible explanation for these relations in our study is that the behavior of parents experiencing psychopathology symptoms differs from parents not facing these difficulties, like displaying more hostile behavior toward their offspring as has been observed elsewhere [13]. The only significant difference between parents of children within the clinical syndrome was found to be total problems where their parents had significantly higher internalizing (ASR scores) as compared to parents with children in the normal range.

However, for the parent-reported syndromes scores (CBCL) we found that mothers were reporting significantly lower scores on externalizing and total problems of their children as compared to fathers (*p* < 0.05). This must be interpreted with caution because the parents were not reporting on shared children (we had only one parent per child) but on different children. There is also the possibility that male parents overreact to externalizing problems which in turn affect the overall total problems, thus agreeing with the observations by Majdandzic et al. 2014 who observed that fathers may take a more firm approach than mothers to children [43].

This study did not investigate the dynamics that explain the frequent association between the two sets of psychopathologies. However, given the majority of similarities between our findings on clinical syndromes on CBCL and severity of ASR syndrome scores and those of the global database on the clinical population of children and parents, the same dynamics that lead to similarities between children and parents psychopathologies may operate in the Kenyan case. It is also possible that parents with psychopathology can easily identify with or magnify pathology in their children. Our findings suggest the possibility that parent and child psychopathology are largely independent i.e. one does not necessarily lead to another. But our findings also suggest that they often co-exist. Thus, a child with psychopathology is likely to have a parent with psychopathology and vice-versa.

This could have influenced their psychopathology as suggested in the literature review. This is particularly relevant in the Kenyan culture where relatives take on the upbringing of their relative’s children in various circumstances. These circumstances include the death of the biological parents, financial constraints preventing parents from raising their children, or physical or mental incapacitation. Additionally, the extended family system, which involves the shared upbringing of children within the community, is still a prevalent cultural practice. However, this cultural practice of the extended family system is increasingly being replaced by the nuclear family system due to the urbanization system – 31% of the Kenyan population live in urban areas [27].

### Limitations

1. We did not explore whether the children we studied were adopted or lived with their biological parents.
2. We did not examine the quality of attachment between the adolescents and their parents – whether biological or not, given that the level of attachment with parents can override the effects of adoption. These limitations can be addressed in future studies including the impact of the new phenomenon of COVID-19. Despite these limitations, we provide a study in a Kenyan setting that contributes to and concurs with the global database on the positive correlation between CBCL and ASR and the general negative association between YSR and CBCL. So far, the preliminary impression is that it is realistic, feasible and acceptable to both parents and children and that this managed dialogue can be conducted by trained, supervised and supported community health workers who ordinarily visit families for many other health-related issues.
3. The presence of unmeasured confounding factors, such as family income and education level, could have impacted the results of the study because these variables can influence various aspects of familial dynamics and child development. There is therefore a need for future research to consider and address a broader range of potential confounding variables to further enhance the validity of the findings.

### Practice implications

Our findings call for a family-centered approach when dealing with children with psychopathology so as to create the opportunity to also identify and manage problems in the parents. This family-centered approach would also attempt to identify more family-related “hidden” factors that may influence the final expression of the children and parents. It would also address more psychosocial and cultural factors and demographic characteristics of the family. This holistic managed dialogue approach creates a win-win situation for both the child and the parents. Further studies in the Kenyan context are required to test this hypothetical possibility and also to determine the actual dynamics that explain the relationship between parent and child psychopathology.

## Data Availability

No datasets were generated or analysed during the current study.
